# Gonococcal Infection: Case Report of Bacteremia and Brief Review of a Series of Cases

**DOI:** 10.7759/cureus.40095

**Published:** 2023-06-07

**Authors:** Mariana Fidalgo, Catarina A Salvado, Francisca Carmo, Pedro C Gil, Margarida Mota

**Affiliations:** 1 Internal Medicine, Centro Hospitalar Vila Nova Gaia/Espinho, Vila Nova Gaia, PRT

**Keywords:** skin lesions, sexually transmitted disease, disseminated gonococcal disease, gonococcal bacteremia, neisseria gonorrhoeae

## Abstract

*Neisseria (N) gonorrhoeae* is the microorganism responsible for the second-most reported sexually transmitted disease in the world, commonly infecting mucosal surfaces such as the endocervix, urethra, and pharynx. Gonococcal disease is generally non-symptomatic or pauci-symptomatic, but if untreated, it can progress to a more serious disease with joint, cardiac, or nervous system involvement. Disseminated gonococcal infection occurs in 0.5 to 3% of patients with gonorrhea and can present with purulent arthritis or a combination of dermatitis, tenosynovitis, and migratory polyarthralgia.

This article presents the case of a 45-year-old woman examined in the emergency room for fever and acute pain in her right shoulder and knee. A few days later, the patient developed petechiae and vesiculopustular lesions on her right hand. Blood analysis showed elevated inflammation markers, and cultures yielded gram-negative diplococcus identified as *N. gonorrhoeae. *The patient was successfully treated with ceftriaxone, with complete remission of signs and symptoms of infection.

The article then examines a series of 42 cases of gonococcal disease diagnosed in a tertiary hospital, their microbiologic susceptibilities, and the antibiotics chosen to treat them.

## Introduction

*Neisseria (N) gonorrhoeae* is a gram-negative bacterium responsible for the second-most reported sexually transmitted infection, gonorrhea [1]. According to the World Health Organization (WHO), *N. gonorrhoeae* caused more than 82.4 million infections in 2020 [1]. Gonococcal disease is generally asymptomatic or mildly symptomatic and manifests as urethritis in men and cervicitis in women. In rare circumstances, the disease can advance to a disseminated gonococcal infection (DGI), presenting as purulent arthritis or a combination of dermatitis, tenosynovitis, and migratory polyarthralgia. It can also lead to endocarditis and central nervous system involvement [2].

## Case presentation

A 45-year-old woman presented to the emergency room (ER) with a fever and acute pain in her right knee and shoulder that started three days prior. The patient´s past medical history included arterial hypertension, dyslipidemia, asymptomatic hyperuricemia, thyroid disease, and depression. She was chronically medicated with nebivolol (5 mg), perindopril + indapamide (5+1.25 mg), atorvastatin (20 mg), fluoxetine (20 mg), and omeprazole (20 mg).

On physical examination, her temperature was 38.4ºC, her blood pressure was 120/71 mmHg, and her heart rate was 100 beats per minute. Active and passive motions of her right shoulder and knee were painful, but neither articulation presented other signs of inflammation. There were no skin lesions. Laboratory tests revealed leukocytosis (12.58x103/uL, with 11.02x103/uL neutrophils), thrombocytosis (437x103/uL), and elevated C-reactive protein (CPR) (3.6 mg/dL). The liver and renal function were normal; urinalysis was unremarkable. Shoulder ultrasonography showed no signs of tenosynovitis, joint effusion, or periarticular abscess. A thoraco-abdominopelvic computed tomography scan was normal. Blood and urine were collected for microbiological cultures and serologic tests. The patient was discharged with acetaminophen and ibuprofen for fever and pain control.

**Figure 1:**
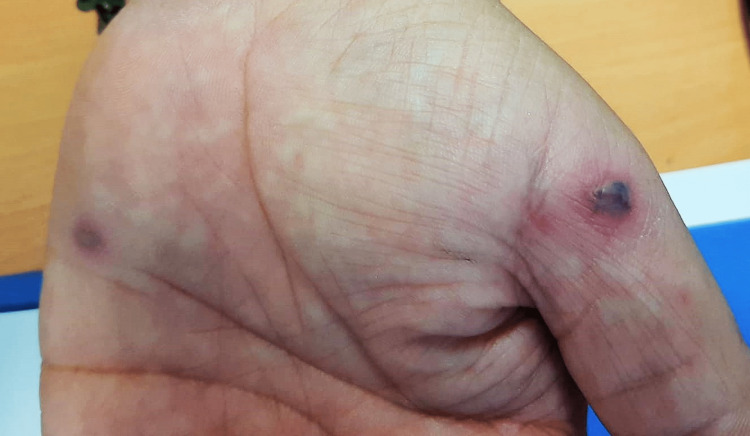
Vesiculopustular lesions on patient’s right hand

**Figure 2:**
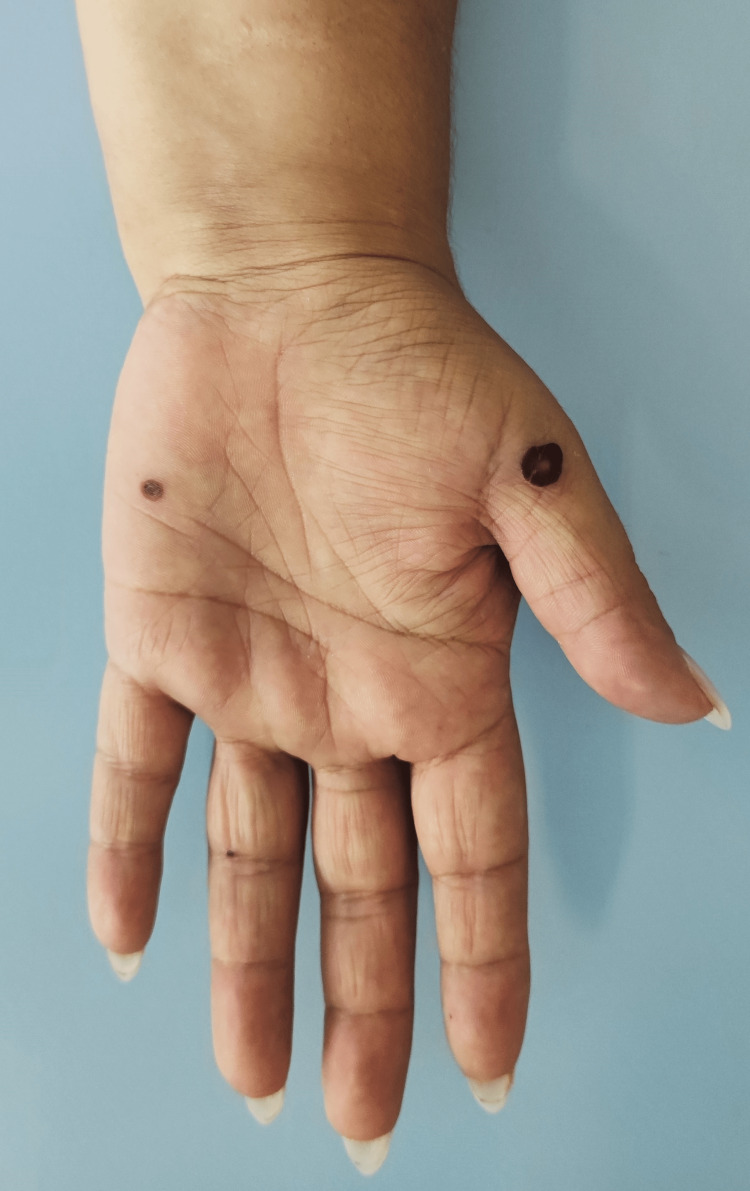
Evolved vesiculopustular lesions on patient’s right hand (one week after)

On a follow-up appointment three days later, the fever and right shoulder pain persisted, and there was right wrist pain as a new symptom. On physical examination, the temperature was 36ºC (under acetaminophen), and the patient reported persistent pain with active and passive movement of the affected joints. As a new sign, there were non-blanchable and non-palpable petechiae and two vesiculopustular lesions on the right hand (Figure 1, 2).

The previously collected blood cultures yielded gram-negative diplococci, with the identification of *Neisseria gonorrhoea*. The patient denied other risk factors for gonorrhea, such as a previously known gonococcal infection or new or multiple sexual partners. The results of a human immunodeficiency virus antibody test, as well as hepatitis B, hepatitis C, cytomegalovirus, Epstein-Barr virus*, Borrelia, Chlamydia trachomatis, Chlamydia pneumoniae, Coxiella burnetti, Rickettsia conorii*, and leptospirosis serologies, were negative.

The patient was started on IV ceftriaxone (2 g/day for seven days) and oral doxycycline (100mg twice daily for seven days as presumptive treatment for chlamydia) at the out-patient clinic, but because of persistent fever, the patient was later admitted for monitoring. She was submitted to a gynecological examination and vaginal and endocervical swabs for nucleic acid amplification testing for *N. gonorrhoeae* (which was positive) and *C. trachomatis* (which was negative). Antimicrobial susceptibility testing was performed on the isolate, showing sensitivity to ceftriaxone, ciprofloxacin, and penicillin. Follow-up blood cultures obtained after 48 hours of ceftriaxone were negative. On the seventh day, the patient was afebrile, with significant arthralgia improvement and normal CPR levels.

## Discussion

Gonococcal bacteremia is a rare condition affecting only 0.5-3% of patients with gonorrhea [2]. The likelihood of hematogenous dissemination depends on host, microbial, and immune factors. Women are at a four-fold higher risk for dissemination than men, especially while menstruating or pregnant [2-3]. Patients with disseminated gonococcal infection rarely have a history of symptomatic genital infection, so asymptomatic infection can be considered a risk factor for dissemination. Complement deficiency and autoimmune disorders such as systemic lupus erythematosus also predispose to a higher risk of disseminated gonococcal infection [3-4].

Clinical manifestations of DGI are usually divided into two classic forms: 1. arthritis-dermatitis syndrome, with the predominance of skin lesions and polyarthralgia; 2. suppurative arthritis. In either presentation, mucosal involvement is rare, even though an oral or genital localized infection (in the urethra, cervix, rectum, or pharynx) precedes the onset of DGI [5]. Skin lesions have an estimated prevalence greater than 40% [2] and are presumably the result of bacterial embolization of the skin with the development of microscopic abscesses. They may appear as petechiae that evolve into papules or pustules, and these can progress to hemorrhagic or necrotic lesions [6].

Our patient is a woman, and she has no clinically evident mucosal involvement, two risk factors for invasive disease. The symptoms of arthralgias and fever, with the later appearance of vesiculopustular skin lesions, suggest DGI in the arthritis-dermatitis form.

Cases of *N. gonorrhoeae* in a tertiary hospital

Between January 2016 and October 2022, 42 cases of *N. gonorrhoeae* were diagnosed in our hospital in nine different biological products. Table 1 contains information provided by the pathology department and comprises all *N. gonorrhoeae* isolates for that period. Twenty-nine (69%) of the subjects were men and 13 (31%) were women, with a mean age of 28 years (min. 8 and max. 61) and a median age of 29 years.

**Table 1:** Cases of N. gonorrhoeae in different biological samples

Biological sample	Number of cases
Urethral exudate	25 (59.5%)
Vaginal exudate	7 (16.7%)
Endocervical exudate	4 (9.5%)
Rectal exudate	2 (4.8%)
Blood	1 (2.4%)
Pharyngeal exudate	1 (2.4%)
Prostatic abscess (pus)	1 (2.4%)
Synovial fluid	1 (2.4%)

Antimicrobial susceptibility tests were available for 39 out of 42 isolates, with all being sensitive to ceftriaxone. Susceptibility to ciprofloxacin was evaluated in 33 of the cases, of which 14 (42%) were sensitive to said drug. The antibiotics chosen for treatment are presented in Table 2.

**Table 2:** Treatment choices Unknown antibiotic choice: treatment was prescribed in a different healthcare facility, and the patient wasn't able to provide the name

Antibiotic	Number of cases
Ceftriaxone	36 (85.7%)
Benzylpenicillin	1 (2.4%)
Cefotaxime	1 (2.4%)
Cefixime	1 (2.4%)
Ciprofloxacin	1 (2.4%)
Unknown	2 (4.8%)

Thirty-five (83.3%) patients with proven or presumed coinfection with *Chlamydia trachomatis* were treated with a single dose of azithromycin or a seven-day course of doxycycline.

*N. gonorrhoeae* has developed resistance to many classes of antibiotics, including penicillins, tetracyclines, macrolides, and fluoroquinolones. Susceptibility data and epidemiologic surveillance programs demonstrate a particularly alarming trend in this bacteria's drug resistance, with a progressive decrease in susceptibility to cephalosporins and azithromycin. Of the cephalosporin class of drugs, ceftriaxone still has the lowest rates of gonococcal drug resistance and, therefore, is recognized as the standard of care [3,7].

Of the 42 cases of microbiological identification of *N. gonorrhoeae*, only two presented as DGI. Both were women; one presented with suppurative arthritis and the other with bacteremia, which is the patient described above. Thus, in the study cohort, 4.8% of cases had disseminated disease, with 2.4% presenting bacteremia. Two of the women with infections identified in vaginal exudate had evidence of a pelvic abscess. These were presumably caused by *N. gonorrhoeae*, as no other cause was identified, and both responded to treatment. However, as the abscesses weren’t drained, no direct microbiological tests were performed, and these remain presumptive diagnoses.

Despite the fact that rates of DGI in our cohort are in accordance with the estimated prevalence numbers, there are some limitations to our study that must be considered: 1) blood cultures weren’t collected in all patients with isolation of *N. gonorrhoeae* in a urethral, vaginal, or endocervical swab, and so bacteremia could be present and undiagnosed; this, however, is unlikely, as none of these patients presented symptoms of disseminated disease; 2) the sample size is small, and conclusions regarding prevalence should be made cautiously.

## Conclusions

Gonococcal infection is common and increasing in frequency throughout the world. Therefore, it is of foremost importance to know its typical and atypical presentations, to identify and treat the infections, and to stop their dissemination. Despite being a rare form of the disease, disseminated gonorrhoea should be suspected in sexually active patients presenting with fever and arthralgia, with or without genitourinary symptoms. When considering a possible sexually transmitted infection (STI), adequate samples should be collected for microbiologic or serologic testing (namely exudates and blood cultures), and screening should include other STIs. Empirical treatment should be promptly started to prevent severe complications and adjusted posteriorly, according to test results.

## References

[1] World Health Organization (2021). Global Progress Report on HIV, Viral Hepatitis and Sexually Transmitted Infections. https://www.who.int/publications/i/item/9789240027077.

[2] Burns JE, Graf EH (2018). The brief case: disseminated Neisseria gonorrhoeae in an 18-year-old female. J Clin Microbiol.

[3] Schweon SJ (2019). Disseminated gonococcal infection. Nursing.

[4] Dutertre M, Tomasevic D, Guillermin Y (2014). Gonococcemia mimicking a lupus flare in a young woman. Lupus.

[5] Blank JA, Thapa N, Mansoor AM (2021). Arthritis-dermatitis syndrome: a case of disseminated gonococcal infection with petechial skin rash. J Gen Intern Med.

[6] Ghosn SH, Kibbi AG (2004). Cutaneous gonococcal infections. Clin Dermatol.

[7] Workowski K, Bachmann L, MD1; Chan P (2021). Centers for Disease Control and Prevention: Sexually Transmitted Infections Treatment Guidelines, 2021. https://www.cdc.gov/std/treatment-guidelines/default.htm.

